# Reactance revisited: Consequences of mandatory and scarce vaccination in the case of COVID‐19

**DOI:** 10.1111/aphw.12285

**Published:** 2021-05-25

**Authors:** Philipp Sprengholz, Cornelia Betsch, Robert Böhm

**Affiliations:** ^1^ Media and Communication Science University of Erfurt Erfurt Germany; ^2^ Center for Empirical Research in Economics and Behavioral Sciences University of Erfurt Erfurt Germany; ^3^ Department of Psychology University of Copenhagen Copenhagen Denmark; ^4^ Department of Economics University of Copenhagen Copenhagen Denmark; ^5^ Copenhagen Center for Social Data Science University of Copenhagen Copenhagen Denmark

**Keywords:** COVID‐19, mandates, psychological reactance, scarcity, vaccination

## Abstract

Psychological reactance theory assumes that the restriction of valued behaviors elicits anger and negative cognitions, motivating actions to regain the limited freedom. Two studies investigated the effects of two possible restrictions affecting COVID‐19 vaccination: the limitation of non‐vaccination by mandates and the limitation of vaccination by scarce vaccine supply. In the first study, we compared reactance about mandatory and scarce vaccination scenarios and the moderating effect of vaccination intentions, employing a German quota‐representative sample (*N* = 973). In the preregistered second study, we replicated effects with an American sample (*N* = 1394) and investigated the consequences of reactance on various behavioral intentions. Results revealed that reactance was stronger when a priori vaccination intentions were low and a mandate was introduced or when vaccination intentions were high and vaccines were scarce. In both cases, reactance increased intentions to take actions against the restriction. Further, reactance due to a mandate was positively associated with intentions to avoid the COVID‐19 vaccination and an unrelated chickenpox vaccination; it was negatively associated with intentions to show protective behaviors limiting the spread of the coronavirus. Opposite intentions were observed when vaccination was scarce. The findings can help policy‐makers to curb the spread of infectious diseases such as COVID‐19.

## INTRODUCTION

The world has waited urgently for vaccines against COVID‐19 to become widely available, potentially stopping the spread of the disease and ending the pandemic. When the first vaccines were in sight, it was discussed in many countries whether vaccination should be mandatory, causing a great deal of criticism (Moorthy, 2020; Prieto Curiel & González Ramírez, 2021). Now that several vaccines have been approved, the halting and insufficient supply of doses has again raised discussions and complaints (Santora, 2021). In this contribution, we ask whether these similarly negative reactions to two quite opposing situations are based on the same psychological mechanism, namely psychological reactance.

Psychological reactance theory (Brehm, 1966) is based on the idea that individuals appreciate behavioral freedom. When freedom is restricted because of a threat or loss of valued behavior, individuals will experience reactance, a composite of anger and negative cognitions (Dillard & Shen, 2005), motivating them to regain the freedom lost. Reactance can manifest in multiple ways. Individuals may be triggered to engage in constrained behavior (boomerang effect), to take action against the restriction, or to preserve other freedoms (Miron & Brehm, 2006).

It has been argued that psychological reactance may account for failures of health campaigns striving to limit harmful behaviors (Byrne & Hart, 2009). Campaigns aiming to decrease vaccine hesitancy by making vaccination mandatory, for example, may elicit reactance and increase, instead of decrease, vaccine hesitancy. Indeed, information about hypothetical mandatory vaccination policies has been shown to elicit reactance, particularly when vaccination intentions and support for mandatory policies were low (Betsch & Böhm, 2016; Sprengholz & Betsch, 2020). Higher reactance, in turn, increased activism (such as intentions to sign a petition against the mandate), as well as tendencies to show fewer protective health behaviors (e.g. wearing masks or avoiding close personal contact during the COVID‐19 pandemic), and decreased intentions to receive other, non‐mandated vaccinations, such as a voluntary flu shot (Sprengholz et al., 2021).

While previous research has investigated the detrimental effects of limiting the freedom to *not* be vaccinated, it is unknown what the consequences are when limiting the freedom to be vaccinated. The COVID‐19 pandemic has created an example of the latter: The initial scarcity of the COVID‐19 vaccines has led to the prioritizing of certain population groups (e.g. health professionals, vulnerable, and older people), while the majority of people will have to wait for months or even years to receive a vaccination (Warren & Lofstedt, 2021). Commodity theory (Brock, 1968) states that the attractiveness of goods increases with their scarcity. That is, when objects, information, or health conditions are rare (Ditto & Jemmott, 1989), the freedom to get them is threatened, which elicits reactance. As a consequence, the motivation to restore the restricted freedom increases, that is leading to actions to gain access to the scarce resource (Brehm, 1972). At the same time, the good's subjective value increases (Worchel et al., 1975).

Therefore, we hypothesised that preferred but scarce vaccination could also trigger reactance and affect behavior to compensate for the restricted freedom. Reactance about vaccine scarcity should increase with people's vaccination intention. Building on psychological reactance theory (Brehm, 1966), reactance due to vaccine scarcity could result in compensatory behavioral intentions opposite from those caused by reactance due to mandatory vaccination. In other words, vaccine scarcity was hypothesised to (i) trigger actions addressing the shortage (e.g. signing a petition) but (ii) reduce intentions to avoid the scarce vaccine and even (iii) increase vaccination intentions against an unrelated disease. For reactance due to a mandatory policy, we expected the opposite effects. We further explored whether scarce or mandatory vaccination changes the willingness to show health protective behaviors. Study 1 explored these effects; Study 2 tested preregistered hypotheses and extended the analysis to the consequences of increased reactance.

## STUDY 1

Study 1 explored the reactance effects of mandatory and scarce vaccination in comparison with an unrestricted vaccination decision, controlling for initial COVID‐19 vaccination intentions.

### Method

#### Participants and design

The experiment was conducted as part of a cross‐sectional survey on December 22 and 23, 2020, and completed by *N* = 973 German participants. The sample was non‐probabilistic and quota‐representative for age × gender and federal state. Participants were 18 to 74 years old (*M* = 44.07, *SD* = 15.25) and included 494 males and 479 females. Most participants (57.6%) had completed secondary education with university entrance qualifications. The experiment implemented a one‐factorial (unrestricted vs. mandatory vs. scarce vaccination) between‐subjects design, with reactance as the dependent variable. Participants’ demographic characteristics did not differ between the experimental conditions (see Supporting information).

#### Materials and measures

The Supporting information provides details on all materials and measures as well as the raw data. COVID‐19 vaccination intentions were assessed before the experimental manipulation took place. Reactance was measured after the experimental manipulation.

##### A priori COVID‐19 vaccination intention

Participants were asked what they would do if they had the opportunity to get a free vaccination against COVID‐19 in the next week. Answers were assessed on 7‐point scales ranging from “I would not get vaccinated at all” to “I would definitely get vaccinated.”

##### Experimental manipulation

Participants were randomly assigned to one of the three conditions. In the unrestricted vaccination condition, participants should imagine that vaccination against COVID‐19 was recommended but voluntary. In the mandatory vaccination condition, they should imagine that the vaccination was mandatory and that non‐compliance would lead to a fine of EUR 2.000 (about USD 2.400). In the scarce vaccination condition, participants were directed to imagine that the vaccine was scarce and that they would have to wait until 2022 if they want to be vaccinated, as elderly people and health professionals would be prioritized.

##### Reactance

An adapted version of the experience of reactance subscale of the Salzburg State Reactance Scale (Sittenthaler et al., 2015) was used. Participants were asked how frustrated, annoyed, and disturbed they felt about the vaccination situation they were told to imagine and whether they perceived it as restricting their freedom. The four items were assessed using a 7‐point scale ranging from 1 (“not at all”) to 7 (“very much”). The mean score was used for all analyses (Cronbach's α = .96).

### Results and discussion

**FIGURE 1:**
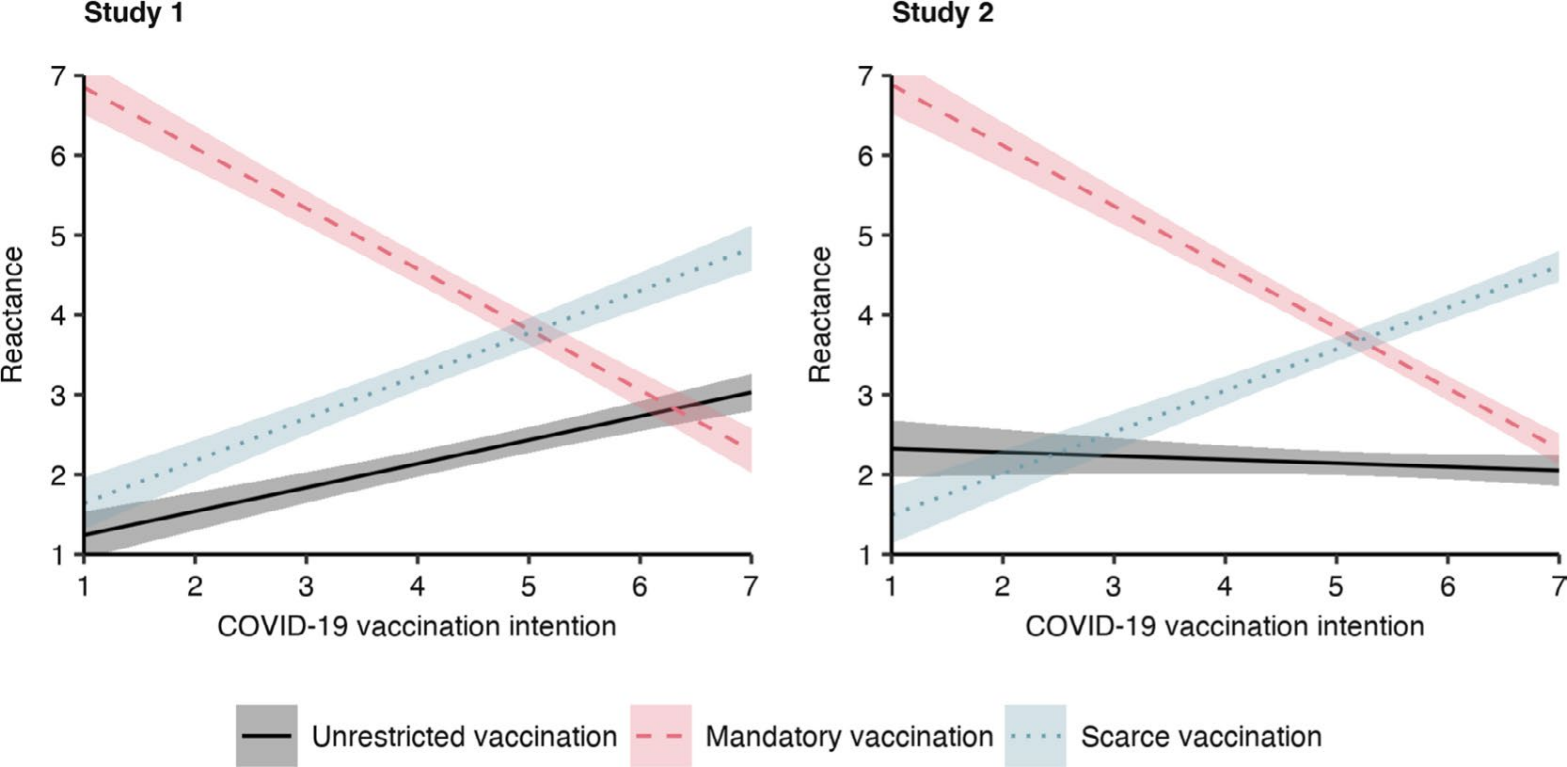
Reactance by COVID‐19 Vaccination Intention and Experimental Manipulation *Note*: Results from linear regression analysis for Studies 1 (Table S1) and 2 (Table S2). Ribbons visualize 95% confidence intervals of predicted values. [Colour figure can be viewed at wileyonlinelibrary.com]

A linear regression analysis was used to investigate the impact of COVID‐19 vaccination intention and experimental conditions (dummy coded with unrestricted vaccination as baseline), as well as their interactions on reactance (Table S1 in the supplement). As Figure 1 shows, reactance increased with stronger vaccination intention, especially in the scarce vaccination condition (interaction: *b* = 0.24; 95% CI [0.13, 0.34]). On the other hand, in the mandatory vaccination condition, the higher the previous vaccination intention, the more reactance decreased (interaction: *b* = −1.06; 95% CI [−1.17, −0.95]).

Thus, as hypothesised, both mandatory and scarce vaccination elicited reactance, particularly in participants whose behavioral intention was affected by the restriction. The finding that reactance became stronger with increasing vaccination intention in the unrestricted vaccination condition may be explained by the fact that most participants probably already knew that COVID‐19 vaccines would be scarce. Since the scenario description focused on voluntariness and did not ask participants to imagine abundant vaccines, the implicit perception of scarcity may have elicited higher levels of reactance when vaccination intentions were high. We addressed this issue in the second study.

## STUDY 2

We aimed to replicate the results in Study 1 that both mandatory and scarce vaccination can elicit reactance. Additionally, we extended the findings to behavioral consequences of reactance (activism against the restriction, avoidance of the vaccine, preservation of other freedoms). The study was preregistered (https://aspredicted.org/23ea4.pdf).

### Method

#### *Participants* *and design*

Data were collected on January 7, 2021, from a US sample recruited via the online recruitment platform Prolific (https://www.prolific.co). The experiment was completed by *n* = 1.701 participants. As preregistered, participants above 60 years of age, health professionals, and those with a chronic condition were excluded as some participants were told that these groups were prioritized for vaccination and thus not affected by scarce supplies, resulting in a final sample of *N* = 1.394 individuals, which is sufficient to find medium‐sized interaction effects in the preregistered regression analyses (*f* ^2^ = 0.15, α = .05, 1–β = .80). Participants in the final sample were 18 to 59 years old (*M* = 33.36, *SD* = 10.16). A total of 564 were female and 69.9% owned a college degree. Most participants were non‐Hispanic White (*n* = 992), followed by participants with Asian (*n* = 163), Black or African American (*n* = 97), and Hispanic or Latino ethnicity (*n* = 87). The experiment implemented a one‐factorial (unrestricted vs. mandatory vs. scarce vaccination) between‐subjects design with reactance as well as various behavioral intentions as dependent variables. Participants’ demographic characteristics did not differ between the experimental conditions (see Supporting Information).

#### *Materials* *and measures*

Details about the materials and measures as well as the raw data can be found in the supplemental online material. Participants were asked about their COVID‐19 vaccination intentions before the experimental manipulation took place. Afterward, reactance and behavioral intentions were assessed.

##### A priori COVID‐19 vaccination intention

Intention was measured as in Study 1.

##### Experimental manipulation

Participants were randomly assigned to one of the three conditions. In all conditions, participants were directed to imagine that vaccination against COVID‐19 was recommended, free of charge, and either unrestricted, mandatory, or scarce, depending on condition. In contrast to the unrestricted vaccination condition of Study 1, we did not only emphasize the voluntariness of vaccination but also the availability of vaccines.

##### Dependent variables

Reactance was measured as in Study 1 (Cronbach's α = .94).

*Activism* was rated as agreement to the statement “I will take action against the scarce [mandatory] vaccination (e.g. by signing a petition, writing to a politician, or taking part in a demonstration)” on a 7‐point scale ranging from “strongly disagree” to “strongly agree.”

*Avoidance* was assessed as ratings of the statement “I will look for ways to avoid a vaccination against COVID‐19” on a 7‐point scale ranging from “strongly disagree” to “strongly agree.”

To test whether participants would take measures to preserve other freedoms after a restriction, we assessed participants’ intention to seek or avoid an unrelated vaccination. Since the study was conducted in the midst of the flu season, we decided to assess intentions for another disease: As (booster) vaccinations against chickenpox are common among adults, participants were directed to imagine that a regular check‐up revealed that they had no antibodies against chickenpox. They were informed that an infection can be severe for adults and that their doctor recommends a free vaccination against chickenpox in this situation. Vaccination intention was recorded using a 7‐point scale ranging from “I would not get vaccinated at all” to “I would definitely get vaccinated.”

For the explorative analyses, participants also rated how often they intend to show certain COVID‐19‐related health behaviors during the next two weeks (wearing a mask when shopping, keeping physical distance in public, avoiding close personal contact, staying home when feeling sick, getting tested for COVID‐19 when feeling sick, entering a positive test result in a tracing app). Each of the six behavioral intentions was measured on a 7‐point scale ranging from “never” to “always,” and an average score was calculated (Cronbach's α = .80).

### Results and discussion

#### Reactance

As in Study 1, we regressed reactance on a priori COVID‐19 vaccination intentions, the experimental conditions (dummy coded with unrestricted vaccination as baseline), and their interactions (Table S2 in the supplement). The results replicate the interaction effects found in Study 1 (Figure 1): The more positive a priori vaccination intentions were the less reactance occurred given mandatory vaccination (interaction: *b* = −0.72; 95% CI [−0.82, −0.61]); on the other hand, stronger a priori intentions were related to increasing reactance given scarce vaccination (interaction: *b* = 0.57; 95% CI [0.46, 0.67]). When vaccination was unrestricted, reactance was low in general and did not relate to COVID‐19 vaccination intention. We assume that emphasizing the availability of free vaccines in the case of unrestricted vaccination eliminated the effect of vaccination intention in this condition, confirming our idea that participants in the same condition of Study 1 may have assumed the vaccine to be scarce based on prior experience.

#### *Behavioral* *intentions*

**FIGURE 2:**
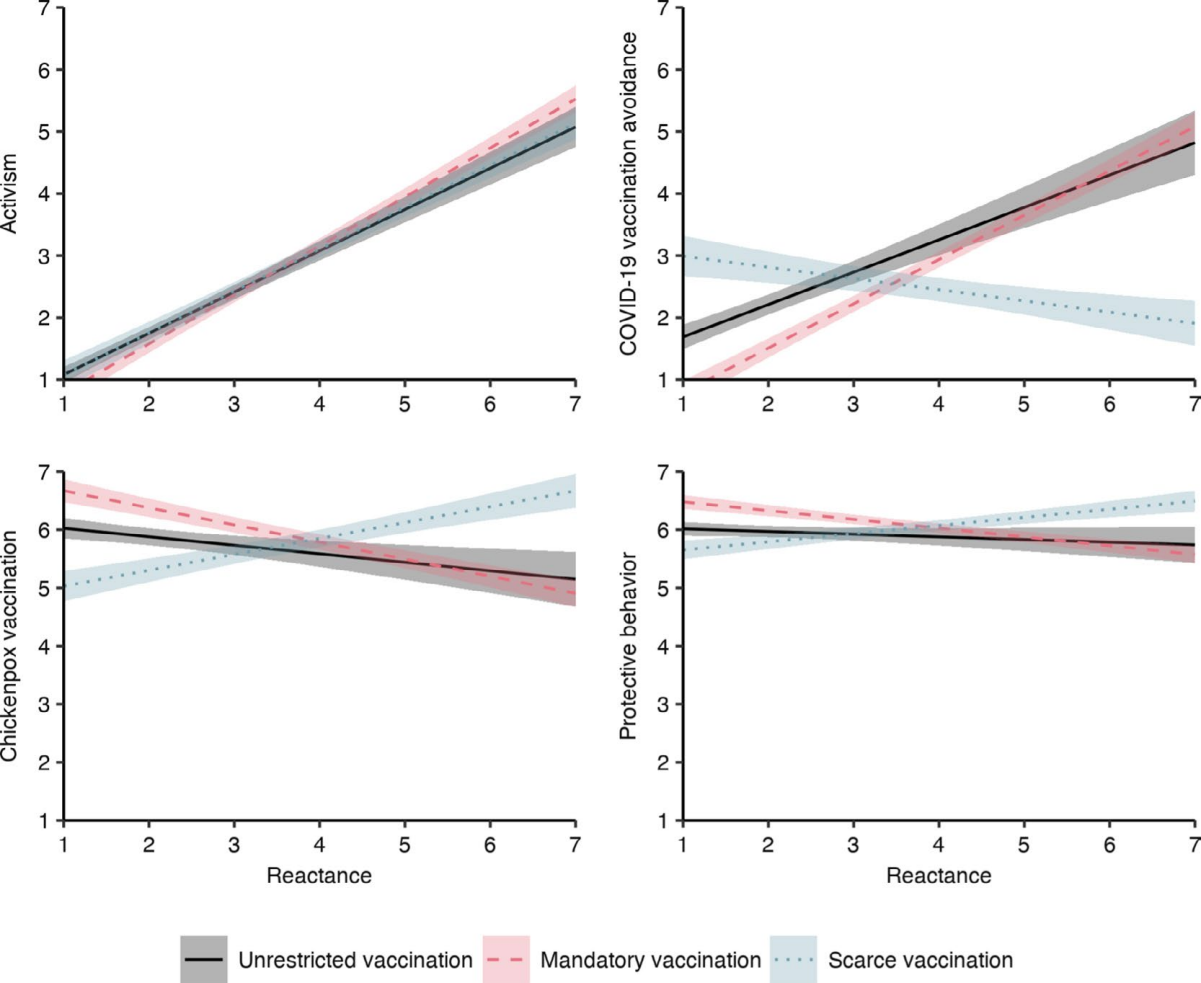
Behavioral Intentions by Reactance and Experimental Manipulation *Note*: Results from linear regression analyses (Table S3). Ribbons visualize 95% confidence intervals of predicted values. [Colour figure can be viewed at wileyonlinelibrary.com]

Linear regression analyses were performed to investigate the effects of reactance, experimental conditions, and their interactions on various behavioral intentions (Figure 2 and Table S3 in the supplement). While higher levels of reactance led to stronger activism intentions (*b* = 0.67; 95% CI [0.59, 0.74]), the effect was stronger when vaccination was mandatory compared to when it was unrestricted (interaction: *b* = 0.12; 95% CI [0.03, 0.22]). The effect did not differ between the unrestricted and scarce vaccination conditions (interaction: *b* = 0.01; 95% CI [−0.09, 0.11]).

Intentions to avoid the COVID‐19 vaccination increased the more reactant people felt; this was especially pronounced in the mandatory vaccination condition (interaction: *b* = 0.19; 95% CI [0.07, 0.32]). Higher reactance led to lower intentions to omit the unrelated vaccination in the scarce vaccination condition (interaction: *b* = −0.70; 95% CI [−0.83, −0.57]).

Regarding the intention to get vaccinated against chickenpox, higher levels of reactance led to stronger chickenpox vaccination intentions when COVID‐19 vaccination was scarce (interaction: *b* = 0.42; 95% CI [0.30, 0.54]) but lower chickenpox vaccination intentions when COVID‐19 vaccination was mandatory (interaction: *b* = −0.15; 95% CI [−0.26, −0.04]).

Finally, the exploratory analysis for COVID‐19‐related health behaviors revealed that when vaccination was scarce, intentions to show protective behaviors increased with increasing reactance (interaction: *b* = 0.19; 95% CI [0.11, 0.26]). When vaccination was mandatory, in contrast, higher levels of reactance were related to lower behavioral intentions (interaction: *b* = −0.11; 95% CI [−0.17, −0.04]). On the level of single behavioral intentions, interaction effects of reactance and mandatory vaccination were strongest for avoiding close personal contact, getting tested for COVID‐19 when feeling sick, and entering a positive test result in a tracing app (Table S4).

In summary, the results confirmed the hypotheses regarding the behavioral consequences of restricting freedom in vaccination decisions: Reactance due to the elimination of a valued choice option—either vaccination or non‐vaccination, depending on the initial vaccination intention—increased intentions to act against that elimination, to seek the eliminated behavior, as well as compensate for the elimination by acting contrary to it in the case of another, non‐mandated vaccination decision. Exploratory evidence further suggests that reactance about mandatory COVID‐19 vaccination decreased the intention to follow recommended protective behaviors, whereas the opposite was true in the case of scarce vaccination. Interestingly, the effects were strongest for the less regulated protective measures (e.g. avoiding close personal contact), indicating that these behaviors are more susceptible to reactance‐based compensation effects than established measures such as mask‐wearing and physical distancing. Overall, reactance effects on chickenpox vaccination and protective behavior intentions were rather small in all experimental conditions. However, even minimal changes in vaccine uptake and adherence to protective measures can have significant effects on the population level, thus being important for public health.

## GENERAL DISCUSSION

Here, we have presented new insights into how opposing situations elicit similar psychological reactions. In line with psychological reactance theory (Brehm, 1966), we showed that two possible limitations of a voluntary vaccination decision, namely eliminating non‐vaccination by mandatory vaccination and eliminating vaccination by scarce vaccine supply, elicit reactance, which in turn motivates behaviors aiming to restore the freedom of choice or compensate for the lack of it.

While our results are theoretically grounded as well as replicate and extend previous empirical research, generalization and application require some caution. Perceptions and intentions in hypothetical scenarios differ from real‐life affects and behaviors (Sheeran, 2002). Yet, it is likely that the effects of mandatory and scarce vaccination are even stronger when the personal freedom of vaccination is at stake in real life, such that our results could be seen as conservative estimates. Further, our findings are based on samples from Germany and the United States and should be replicated in other countries. Since vaccination mandates are common school entry and employment criteria in the United States, stronger reactance effects could be found in other countries where mandates are less common.

Overall, our results indicate detrimental effects of COVID‐19 vaccination mandates on important other health behaviors; for individuals with low a priori vaccination intentions, mandating vaccination could lead to less uptake of other, still voluntary vaccines and reduce application of protective measures preventing the spread of the coronavirus. In line with previous research (Sprengholz et al., 2021), announcing a future mandate could fuel current disease dynamics and impede a country's vaccination program. For instance, mandatory COVID‐19 vaccination could lead to lower vaccination intentions for flu shots, possibly resulting in worse seasonal influenza epidemics that threaten health systems already burdened with handling the COVID‐19 pandemic. In case policy‐makers decide for the introduction of vaccination mandates, the reasons for doing so should be clearly communicated. Previous research indicates that reactance can be reduced considerably when individuals are informed about the benefits of high vaccination rates, both for public health and for the economy (Sprengholz et al., 2021). As current research suggests that vaccination against COVID‐19 prevents transmission of the disease (Levine‐Tiefenbrun et al., 2021), communicating this prosocial effect of vaccination can also help to leverage support for mandates (Korn et al., 2021; Sprengholz & Betsch, 2020).

Scarce vaccination was related to more favorable behavioral intentions and seemed to increase the attractiveness of the vaccine. While vaccine shortages complicate the fight against COVID‐19, they also seem to foster individual protective behaviors. Consequently, if vaccines are scarce, this should be clearly communicated to the public. People should not be misled into believing that they will soon receive a vaccination when supplies are short as this could jeopardize adherence to protective measures.

It is beyond the scope of this research to evaluate the ethical queries related to the implementation and communication of mandatory or scarce vaccination. However, our findings may help social scientists and policy‐makers in understanding the psychological effects associated with different vaccination policies and communication strategies and thus help curb the spread of infectious diseases such as COVID‐19.

## ETHICAL APPROVAL STATEMENT

Our research obtained ethical clearance from the University of Erfurt's IRB (#20200302/20200501), and all participants provided informed consent prior to data collection.

## CONFLICTS OF INTEREST

The authors declare that they have no conflicts of interest.

## Supporting information

Table S1‐S4

## Data Availability

The materials, data, and data analysis scripts, as well as supplemental tables, are available at: https://dx.doi.org/10.17605/OSF.IO/KTP98 [link for peer review].
