# Supplementary material for: Reactance revisited: Consequences of mandatory and scarce vaccination in the case of COVID‐19
Source: Appl Psychol Health Well Being. 2021 May 25;13(4):986–95. doi: 10.1111/aphw.12285 (PMC8239828; doi:10.1111/aphw.12285)
Supplement: Supplementary file 1 — Table S1‐S4 [file APHW-13-986-s001.docx]

**Supplement**

**Table S1**

COVID-19 vaccination intention and experimental manipulation predicting reactance in Study 1

| Predictor | *β* | *b* | *SE* | *CI-* | *CI+* |
| --- | --- | --- | --- | --- | --- |
| (Constant) |  | 2.27 | 0.09 | 2.091 | 2.446 |
| Vaccination intention | 0.31 | 0.30 | 0.04 | 0.220 | 0.376 |
| Experimental manipulation |  |  |  |  |  |
| Mandatory vaccination (vs. unrestricted vaccination) | 0.42 | 1.97 | 0.13 | 1.718 | 2.218 |
| Scarce vaccination (vs. unrestricted vaccination) | 0.26 | 1.21 | 0.13 | 0.959 | 1.457 |
| Interactions |  |  |  |  |  |
| Vaccination intention × mandatory vaccination | -0.63 | -1.06 | 0.06 | -1.166 | -0.947 |
| Vaccination intention × scarce vaccination | 0.14 | 0.24 | 0.06 | 0.125 | 0.344 |

*Note.* *R*^2^ = .46, adjusted *R*^2^ = .46. All predictors were statistically significant with *p* < .05. *CI-* and *CI+* are the lower and upper bonds of the 95% confidence interval. Vaccination intention was mean-centered before analysis.

**Table S2**

COVID-19 vaccination intention and experimental manipulation predicting reactance in Study 2

| Predictor | *β* | *b* | *SE* | *CI-* | *CI+* |
| --- | --- | --- | --- | --- | --- |
| (Constant) |  | **2.13** | 0.08 | 1.976 | 2.278 |
| Vaccination intention | -0.04 | -0.05 | 0.04 | -0.122 | 0.031 |
| Experimental manipulation |  |  |  |  |  |
| Mandatory vaccination (vs. unrestricted vaccination) | **0.33** | **1.46** | 0.11 | 1.249 | 1.669 |
| Scarce vaccination (vs. unrestrcited vaccination) | **0.37** | **1.62** | 0.11 | 1.407 | 1.831 |
| Interactions |  |  |  |  |  |
| Vaccination intention × mandatory vaccination | **-0.40** | **-0.72** | 0.05 | -0.822 | -0.609 |
| Vaccination intention × scarce vaccination | **0.32** | **0.57** | 0.05 | 0.459 | 0.671 |

*Note.* *R*^2^ = .39, adjusted *R*^2^ = .39. Bold values are statistically significant with *p* < .05. *CI-* and *CI+* are the lower and upper bonds of the 95% confidence interval. Vaccination intention was mean-centered before analysis.

**Table S3**

Reactance and experimental manipulation predicting behavior intentions.

| Dependent variable and predictors | *β* | *b* | *SE* | *CI-* | *CI+* |
| --- | --- | --- | --- | --- | --- |
| (1) Activism (*R*^2^ = .57, adjusted *R*^2^ = .57) | | | | | |
| (Constant) |  | **2.53** | 0.07 | 2.383 | 2.669 |
| Reactance | **0.70** | **0.67** | 0.04 | 0.590 | 0.740 |
| Experimental manipulation |  |  |  |  |  |
| Mandatory vaccination (vs. unrestricted vaccination) | -0.01 | -0.03 | 0.09 | -0.210 | 0.161 |
| Scarce vaccination (vs. unrestricted vaccination) | 0.01 | 0.02 | 0.09 | -0.169 | 0.209 |
| Interactions |  |  |  |  |  |
| Reactance × mandatory vaccination | **0.08** | **0.12** | 0.05 | 0.033 | 0.215 |
| Reactance × scarce vaccination | 0.01 | 0.01 | 0.05 | -0.087 | 0.106 |
| (2) COVID-19 vaccination avoidance (*R*^2^ = .28, adjusted *R*^2^ = .28) | | | | | |
| (Constant) |  | **2.82** | 0.10 | 2.629 | 3.014 |
| Reactance | **0.53** | **0.52** | 0.05 | 0.421 | 0.623 |
| Experimental manipulation |  |  |  |  |  |
| Mandatory vaccination (vs. unrestricted vaccination) | **-0.11** | **-0.48** | 0.13 | -0.726 | -0.227 |
| Scarce vaccination (vs. unrestricted vaccination) | -0.05 | -0.22 | 0.13 | -0.475 | 0.034 |
| Interactions |  |  |  |  |  |
| Reactance × mandatory vaccination | **0.13** | **0.19** | 0.06 | 0.069 | 0.315 |
| Reactance × scarce vaccination | **-0.40** | **-0.70** | 0.07 | -0.833 | -0.573 |
| (3) Chickenpox vaccination (*R*^2^ = .10, adjusted *R*^2^ = .10) | | | | | |
| (Constant) |  | **5.71** | 0.09 | 5.537 | 5.880 |
| Reactance | **-0.19** | **-0.15** | 0.05 | -0.236 | -0.056 |
| Experimental manipulation |  |  |  |  |  |
| Mandatory vaccination (vs. unrestricted vaccination) | **0.10** | **0.33** | 0.11 | 0.103 | 0.548 |
| Scarce vaccination (vs. unrestricted vaccination) | -0.02 | -0.09 | 0.12 | -0.312 | 0.142 |
| Interactions |  |  |  |  |  |
| Reactance × mandatory vaccination | **-0.12** | **-0.15** | 0.06 | -0.258 | -0.039 |
| Reactance × scarce vaccination | **0.30** | **0.42** | 0.06 | 0.304 | 0.536 |
| (4) COVID-19 related protective behaviors (*R*^2^ = .07, adjusted *R*^2^ = .07) | | | | | |
| (Constant) |  | **5.92** | 0.06 | 5.808 | 6.024 |
| Reactance | -0.09 | -0.05 | 0.03 | -0.103 | 0.011 |
| Experimental manipulation |  |  |  |  |  |
| Mandatory vaccination (vs. unrestricted vaccination) | **0.11** | **0.24** | 0.07 | 0.097 | 0.377 |
| Scarce vaccination (vs. unrestricted vaccination) | 0.02 | 0.04 | 0.07 | -0.102 | 0.184 |
| Interactions |  |  |  |  |  |
| Reactance × mandatory vaccination | **-0.14** | **-0.11** | 0.04 | -0.174 | -0.036 |
| Reactance × scarce vaccination | **0.21** | **0.19** | 0.04 | 0.113 | 0.259 |

*Note.* Bold values are statistically significant with *p* < .05. *CI-* and *CI+* are the lower and upper bonds of the 95% confidence interval. Reactance was mean-centered before analysis.

**Table S4**

Reactance and experimental manipulation predicting protective behaviors in detail

| Dependent variable and predictors | *β* | *b* | *SE* | *CI-* | *CI+* |
| --- | --- | --- | --- | --- | --- |
| (1) Wearing mask when shopping (*R*^2^ = .04, adjusted *R*^2^ = .04) | | | | | |
| (Constant) |  | **6.49** | 0.06 | 6.378 | 6.597 |
| Reactance | **-0.25** | **-0.12** | 0.03 | -0.180 | -0.065 |
| Experimental manipulation |  |  |  |  |  |
| Mandatory vaccination (vs. unrestricted vaccination) | 0.07 | 0.14 | 0.07 | -0.000 | 0.284 |
| Scarce vaccination (vs. unrestricted vaccination) | 0.03 | 0.07 | 0.07 | -0.076 | 0.214 |
| Interactions |  |  |  |  |  |
| Reactance × mandatory vaccination | -0.00 | -0.00 | 0.04 | -0.072 | 0.068 |
| Reactance × scarce vaccination | **0.20** | **0.18** | 0.04 | 0.102 | 0.250 |
| (2) Physical distancing in public (*R*^2^ = .03, adjusted *R*^2^ = .03) | | | | | |
| (Constant) |  | **6.16** | 0.06 | 6.034 | 6.280 |
| Reactance | -0.11 | -0.06 | 0.03 | -0.124 | 0.005 |
| Experimental manipulation |  |  |  |  |  |
| Mandatory vaccination (vs. unrestricted vaccination) | **0.08** | **0.19** | 0.08 | 0.026 | 0.346 |
| Scarce vaccination (vs. unrestricted vaccination) | 0.04 | 0.10 | 0.08 | -0.065 | 0.261 |
| Interactions |  |  |  |  |  |
| Reactance × mandatory vaccination | -0.07 | -0.06 | 0.04 | -0.141 | 0.016 |
| Reactance × scarce vaccination | **0.17** | **0.16** | 0.04 | 0.080 | 0.247 |
| (3) Avoiding close contacts (*R*^2^ = .04, adjusted *R*^2^ = .04) | | | | | |
| (Constant) |  | **5.87** | 0.08 | 5.719 | 6.024 |
| Reactance | -0.10 | -0.06 | 0.04 | -0.144 | 0.016 |
| Experimental manipulation |  |  |  |  |  |
| Mandatory vaccination (vs. unrestricted vaccination) | **0.08** | **0.23** | 0.10 | 0.030 | 0.426 |
| Scarce vaccination (vs. unrestricted vaccination) | 0.01 | 0.03 | 0.10 | -0.174 | 0.230 |
| Interactions |  |  |  |  |  |
| Reactance × mandatory vaccination | **-0.10** | **-0.11** | 0.05 | -0.203 | -0.009 |
| Reactance × scarce vaccination | **0.17** | **0.21** | 0.05 | 0.102 | 0.308 |
| (4) Staying home when feeling sick (*R*^2^ = .02, adjusted *R*^2^ = .02) | | | | | |
| (Constant) |  | **6.46** | 0.05 | 6.364 | 6.555 |
| Reactance | **-0.26** | **-0.11** | 0.03 | -0.157 | -0.057 |
| Experimental manipulation |  |  |  |  |  |
| Mandatory vaccination (vs. unrestricted vaccination) | **0.10** | **0.18** | 0.06 | 0.055 | 0.303 |
| Scarce vaccination (vs. unrestricted vaccination) | **0.07** | **0.13** | 0.07 | 0.007 | 0.260 |
| Interactions |  |  |  |  |  |
| Reactance × mandatory vaccination | 0.08 | 0.05 | 0.03 | -0.008 | 0.113 |
| Reactance × scarce vaccination | **0.16** | **0.12** | 0.03 | 0.054 | 0.183 |
| (5) Getting tested for COVID-19 when feeling sick (*R*^2^ = .05, adjusted *R*^2^ = .05) | | | | | |
| (Constant) |  | **5.42** | 0.10 | 5.234 | 5.609 |
| Reactance | -0.03 | -0.03 | 0.05 | -0.125 | 0.072 |
| Experimental manipulation |  |  |  |  |  |
| Mandatory vaccination (vs. unrestricted vaccination) | **0.11** | **0.41** | 0.12 | 0.162 | 0.649 |
| Scarce vaccination (vs. unrestricted vaccination) | 0.02 | 0.05 | 0.13 | -0.194 | 0.303 |
| Interactions |  |  |  |  |  |
| Reactance × mandatory vaccination | **-0.13** | **-0.17** | 0.06 | -0.285 | 0.045 |
| Reactance × scarce vaccination | **0.07** | **0.29** | 0.07 | 0.159 | 0.412 |
| (6) Entering positive test result in tracing app (*R*^2^ = .05, adjusted *R*^2^ = .05) | | | | | |
| (Constant) |  | **5.10** | 0.11 | 4.879 | 5.323 |
| Reactance | 0.11 | 0.11 | 0.06 | -0.111 | 0.222 |
| Experimental manipulation |  |  |  |  |  |
| Mandatory vaccination (vs. unrestricted vaccination) | 0.07 | 0.28 | 0.15 | -0.007 | 0.569 |
| Scarce vaccination (vs. unrestricted vaccination) | -0.03 | -0.14 | 0.15 | -0.430 | 0.158 |
| Interactions |  |  |  |  |  |
| Reactance × mandatory vaccination | **-0.22** | **-0.35** | 0.07 | -0.489 | 0.205 |
| Reactance × scarce vaccination | **0.09** | **0.17** | 0.08 | 0.017 | 0.317 |

*Note.* Bold values are statistically significant with *p* < .05. *CI-* and *CI+* are the lower and upper bonds of the 95% confidence interval. Reactance was mean-centered before analysis.
